# Functional Dyspepsia

**Published:** 1911-05-06

**Authors:** Allan B. Fearnley


### The General Practitioner's Column.

[Contributions to this Column are invited, and, if accepted, will be paid for.]

### FUNCTIONAL DYSPEPSIA.

By ALLAN B. FEARNLEY, M.B.

It is said of an eminent physician, now deceased, that his advice to his students on the subject of the use of drugs in dyspepsia was as follows: "Gentlemen, when you fail with your alkalies try with your acids," and this sums up the attitude with which the general practitioner is only too prone to regard the treatment of this common complaint. Familiarity breeds contempt, and although it is admitted that a wide clinical experience is essential for the acquirement of an accurate knowledge of any disease, yet such a condition as dyspepsia, which every practitioner has ample opportunities for studying every day, and thereby building up a sound clinical experience, is neglected, and merely treated empirically—groping in the dark, instead of being conscientiously studied.

This attitude is probably accounted for by the confusion of thought which arises from the necessarily inadequate teaching devoted to the subject in hospitals, producing a feeling of despair of ever arriving at any accurate knowledge of the true causes of the complaint, and, therefore, of its successful diagnosis and treatment.

### An Attempt at Classification.

Yet in spite of the obvious difficulties of a diagnosis, which must to a large extent rest on subjective symptoms described with more or less inaccuracy by the patient, and despite the absence of practical and reliable clinical methods, it is nevertheless quite possible to form a perfectly definite and simple scheme of tabulation into which practically all cases of purely functional dyspepsia will fall. It must be clearly understood that such a scheme is only a "working plan," founded on general principles, not pretending to be scientifically accurate in the absence of scientifically accurate data—not dogmatic, but adjustable to each individual case.

To begin with, then, the old classifications which are still to be found, even in some recent text-books, are best forgotten—renal, flatulent, nervous, irritative, bilious, etc. For after excluding dyspepsias secondary to organic disease and to general diseases such as phthisis, morbus cordis, cirrhosis, the gastric crises, etc., there are two types of functional dyspepsia, and only two—(*a*) the hypertonic type, characterised by an excessive secretion of hydrochloric acid and probably also in most cases an increase in motility causing more or less cramp or spasm, and (*b*) the atonic type associated with a diminished acid secretion and certainly by a decrease in motility.

This classification will be found practically to cover all cases of purely functional dyspepsia. Every case should be approached on these lines and all questions asked and examinations conducted with the object of deciding to which of those two classes the case belongs.

On what, then, does the diagnosis rest? Firstly, there are two fairly definite types of patient corresponding to the two classes described.

The hypertonic is typically otherwise robust, with large appetite often athletic, a meat eater, and seldom an abstainer, what may be called the "positive" type, and, a most important point, with a high-tension slow pulse. Pain is his most pronounced symptom. Whereas the typical atonic is the "negative" type, flabby, tired, often anæmic, with nausea in place of appetite, and a low-tension pulse, complaining of discomfort and flatulence rather than pain. Both are almost invariably constipated.

With regard to symptoms, any dogmatic statement is dangerous, since in practice the two types merge into one, except in one respect, which, if it is marked, determines the diagnosis—namely, time of onset of the symptoms.

### The Hypertonic Type.

In the hypertonic dyspeptic, acid probably continues to be poured out after all the food has passed the pylorus, that is to say, the symptoms begin after digestion has ceased, some hours after food, and this also explains the fact that these cases are often relieved by taking food, so that the patient may ascribe all his symptoms to hunger.

There are two other symptoms which, when they appear, are characteristic of the hypertonic form—namely, heartburn and pyrosis—the gushing of alkaline fluid from the mouth being presumably an attempt of nature to deal with the trouble by the secretion of a super-alkaline saliva.

In the atonic form the lack of acid is probably always associated with decreased motility; the food remaining too long in the organ, becomes, as it were, a foreign body, and calls forth a profuse mucous secretion from the stomach, which probably never becomes properly emptied and clean; therefore the symptoms appear as soon as the food enters the stomach and remain more or less intense until the time for the next meal.

Occasionally home remedies which the patient has tried may help in diagnosis. Partial relief obtained from bicarbonate of soda points to the hypertonic form; from acid fruits, such as oranges, to the atonic. Sometimes no definite diagnosis can be made till the effect of drugs has been tried. Under such circumstances it is always wise to treat all doubtful cases as atonic; since an error in this direction causes less discomfort to the patient than the exhibition of acids would in the hypertonic form.

### Treatment.

In the treatment of dyspepsia there are two indications common to both varieties—(*a*) attention to the teeth where necessary, and (*b*) correction of constipation. Apart from this the treatment of each variety is distinct.

General Treatment consists of rest in bed for a few days in aggravated cases, and, where possible, in freedom from mental worry, overwork (especially where associated with hurried mastication), and removal from uncongenial surroundings.

In the hypertonic form hot baths and Turkish baths do good in promoting excretion from the skin, since it is probable that the condition is associated with some kind of auto-intoxication by absorption from the intestine. Vigorous exercise is also indicated in these cases for the same reason.

In the atonic type, abdominal massage and graduated exercises, such as are used in ordinary chlorosis, are useful.

### Diet.

In the causation of functional dyspepsia, diet probably has much less to do than would appear at first sight, with a few exceptions. For instance, it is probable that excessive meat-eating, especially when combined with a free consumption of alcohol, and when associated with lack of sufficient exercise, predisposes to the hypertonic form and to the high arterial tension associated with it, while common salt probably assists in the production of the acid in the gastric juice, and condiments—mustard, etc.—stimulate its excessive secretion. Therefore in the dietetic treatment of dyspepsia, where there is no actual gastritis present, the indications are few—limitation of meat and alcohol in hypertonic cases, with substitution of boiled meat for roast and exclusion of salt and condiments, and in atonic cases a "dry" diet with exclusion of green vegetables, which aggravate flatulence and addition of alcohol daily to one of the meals. By a "dry" diet is meant avoidance of "slops," which again tend to increase flatulence, and the substitution of dried bread or hard dry toast for ordinary soft bread.

### Drugs.

The first indication is the treatment of the constipation, and for this the best aperient in all cases to begin with is calomel. In hypertonic cases associated with high-tension pulse, its use may be continued for a week in fractional doses (gr. $\frac{1}{6}$-$\frac{1}{4}$) every night, assisted by dram doses of sodium sulphate in half a pint of water every morning. It is a detail of some importance to withhold natural mineral waters in these cases, inasmuch as they contain considerable quantities of sodium chloride, and are therefore indicated in the atonic form, though here calomel should not be continued, being replaced by some such drug as cascara.

Coming to drugs which are intended to act directly upon the stomach, the important point is that they must be administered after food; if they are to modify the course of digestion they must be given during digestion. Administration of alkalies before meals, with or without the bitter infusions, is good, but simply from the point of view of lavage; and is therefore equally good in either variety, and to be most effective must be given in half a pint of water.

### Indications for Alkalies.

In the hypertonic cases, then, the obvious cry is for alkalies, the best being sod. bicarb. and bismuth carbonate in combination, and given in large doses. Probably it is useless to give less than 20 gr. of the bismuth salt as a dose, or, if the fluid form is used, the B.P. liq. bismuth et ammon. cit.—using 2 drachms as a minimum dose—with either a sedative such as HCN may be combined. Bismuth is also administered in the form of lozenges given when the pain comes on. These probably act mechanically to a certain extent at least, causing an increased flow of alkaline saliva, since the amount of bismuth in them is too small to have much effect, and also the pain may be to some extent relieved by sucking any hard substance, such as a pebble. If there is any gouty tendency, guaiacol may be separately administered in cachets.

### Acids.

In atonic cases the important drug is hydrochloric acid. It must be remembered in prescribing HCl that in many of these cases the gastric mucosa is in an irritable condition, and may therefore need the addition of morphia in the form of liq. morphine hydrochlor to enable it to stand the acid. The morphia salt should, of course, be withdrawn as soon as possible. The mixture may be made up with a bitter and a carminative, such as peppermint or ginger; sometimes pepsin may be added, though its advantages seem to be disputed.

### After-Treatment.

It must be remembered that when the acute stage has passed off the patient still has the tendency to relapse as soon as treatment is stopped, because the dyspepsia is merely the symptom of an underlying cause. Most cases of hypertonic dyspepsia are associated with a raised arterial tension tending to actual arterio-sclerosis, and in these a partial vegetarian diet should be advised, with considerable restriction of alcohol, and in the more intractable cases a more or less prolonged course of iodides with potass. citrate and an occasional short course of fractional doses of calomel In the atonic form the underlying cause is debility, usually associated with some such condition as convalescence from acute illness, prolonged mental worry, or overwork, and often with anæmia.

It will be found in actual practice that diagnosis and treatment are often not quite so straightforward and simple as they appear upon paper. It must be remembered that we are depending almost entirely upon subjective symptoms for diagnosis, and that patients are apt to exaggerate and make misleading statements. There are certainly some cases where no organic lesion can be discovered, and yet they cannot be made to fall under either of the classes described, as, for instance, the true neuralgia of the stomach, known as gastralgia; but nevertheless it will be found that by approaching cases of functional dyspepsia on some such lines as those indicated there will be fewer disappointments both for patient and doctor.

